# PILS proteins provide a homeostatic feedback on auxin signaling output

**DOI:** 10.1242/dev.200929

**Published:** 2022-07-12

**Authors:** Elena Feraru, Mugurel I. Feraru, Jeanette Moulinier-Anzola, Maximilian Schwihla, Jonathan Ferreira Da Silva Santos, Lin Sun, Sascha Waidmann, Barbara Korbei, Jürgen Kleine-Vehn

**Affiliations:** 1 Institute of Molecular Plant Biology (IMPB), Department of Applied Genetics and Cell Biology, University of Natural Resources and Life Sciences, Vienna (BOKU), Muthgasse 18, 1190 Vienna, Austria; 2 Faculty of Biology, Department of Molecular Plant Physiology (MoPP), University of Freiburg, 79104 Freiburg, Germany; 3 Center for Integrative Biological Signalling Studies (CIBSS), University of Freiburg, 79104 Freiburg, Germany

**Keywords:** GASP1, PILS proteins, RING proteins, Auxin feedback, Auxin signaling, Forward genetic screen, *Arabidopis*

## Abstract

Multiple internal and external signals modulate the metabolism, intercellular transport and signaling of the phytohormone auxin. Considering this complexity, it remains largely unknown how plant cells monitor and ensure the homeostasis of auxin responses. PIN-LIKES (PILS) intracellular auxin transport facilitators at the endoplasmic reticulum are suitable candidates to buffer cellular auxin responses because they limit nuclear abundance and signaling of auxin. We used forward genetics to identify *gloomy and shiny pils* (*gasp*) mutants that define the PILS6 protein abundance in a post-translational manner. Here, we show that *GASP1* encodes an uncharacterized RING/U-box superfamily protein that impacts on auxin signaling output. The low auxin signaling in *gasp1* mutants correlates with reduced abundance of PILS5 and PILS6 proteins. Mechanistically, we show that high and low auxin conditions increase and reduce PILS6 protein levels, respectively. Accordingly, non-optimum auxin concentrations are buffered by alterations in PILS6 abundance, consequently leading to homeostatic auxin output regulation. We envision that this feedback mechanism provides robustness to auxin-dependent plant development.

## Abstract (precis)

**Summary:** Auxin exerts a posttranslational feedback regulation on the PILS proteins, contributing to cellular auxin homeostasis and providing robustness to plant growth and development.

## INTRODUCTION

More than a century of intensive auxin research revealed the crucial role of auxin signaling for growth and developmental processes, such as embryogenesis, root and shoot development, reproductive development and plant responses to the environment (Weijers and Wagner, 2016; Weijers et al., 2018; Gallei et al., 2020; Pernisova and Vernoux, 2021; Anfang and Shani, 2021). Auxin activity is dependent on a variety of auxin transporters that modulate the direction of auxin flow, which is crucial for establishing auxin gradients and for keeping the intracellular auxin homeostasis. Among the most prominent transporters are the PIN-FORMED (PIN) proteins that transport auxin from cell to cell (known as intercellular auxin transport) or across the endoplasmic reticulum (ER) membrane (known as intracellular auxin transport) (Adamowski and Friml, 2015; Zwiewka et al., 2019). The role of the PIN proteins with a long intracellular loop (called canonical) is well established. They localize at the plasma membrane (PM) and define the polar auxin transport that establishes gradients and modulates a plethora of growth and developmental processes (Adamowski and Friml, 2015; Zwiewka et al., 2019). In contrast to the canonical PINs, the developmental roles of the PIN proteins with a short intracellular loop, such as PIN5 and PIN8 (called non-canonical), or with an intermediate loop, such as PIN6 (called semi-canonical), are less understood. These non-canonical auxin transporters localize to the ER (Mravec et al., 2009; Dal Bosco et al., 2012; Ding et al., 2012; Sawchuk et al., 2013; Ganguly et al., 2014; Simon et al., 2016; Ditengou et al., 2018). The PIN-LIKES (PILS) are predicted to be structurally similar to the PIN proteins and belong to another family of putative auxin transporters at the ER (Barbez et al., 2012). The PILS and non-canonical PIN proteins may perform a similar function in transporting auxin at the ER but, importantly, they evolved independently, at least in the plant lineage (Feraru et al., 2012; Bogaert et al., 2022). The PIN5- and PILS-based compartmentalization of auxin correlates with higher auxin conjugation rates (Mravec et al., 2009; Barbez et al., 2012; Dal Bosco et al., 2012; Ding et al., 2012). Auxin conjugates are thought to play important roles as storage forms for the active plant hormone indole-3-acetic acid (IAA) and, hence, intracellular auxin transport at the ER was suspected to play a role in cellular auxin homeostasis (Mravec et al., 2009; Barbez et al., 2012; Dal Bosco et al., 2012; Ding et al., 2012). On the other hand, PILS proteins define the nuclear abundance of auxin, presumably by limiting auxin diffusion from the cytosol into the nucleus, which affects the differential (asymmetric) growth during apical hook development as well as the overall shoot and root organ growth rates (Barbez et al., 2012; Beziat et al., 2017; Feraru et al., 2019). Moreover, PILS proteins integrate internal and environmental signals, such as brassinosteroid, light and temperature, into auxin-dependent growth programs (Beziat et al., 2017; Feraru et al., 2019; Sun et al., 2020), arguing against a purely homeostatic role.

In this article, we introduce a forward genetic screen that identified *gloomy and shiny pils* (*gasp*) mutants that define PILS6 protein abundance. We found that *GASP1* encodes a non-characterized E3 ubiquitin ligase from the RING/U-box family. *gasp1* mutants show altered auxin signaling output, which appears to be compensated by altered PILS6 abundance at the ER membrane. Our data propose that auxin exerts a posttranslational feedback regulation on the PILS proteins, thereby contributing to cellular auxin homeostasis.

## RESULTS

### Forward genetic screen for potential regulators of PILS6

**Fig. 1.**
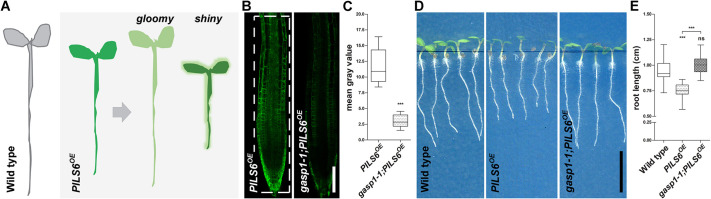
**Forward genetic screen for GASP regulators of PILS6 function.** (A) Graphical representation of the forward genetic screen performed for the identification of *gasp* mutants. EMS-mutagenized seedlings of *PILS6^OE^* were grown for 4 days under standard growth conditions of 21°C and subsequently transferred for 24 h to moderately high temperature (29°C). The seedlings showing either weaker (gloomy) or stronger (shiny) PILS6-GFP signal than *PILS6^OE^* were selected, confirmed and identified as *gasp* mutants of *PILS6^OE^*. Overall, the gloomy and shiny mutants displayed enhanced and reduced growth when compared with *PILS6^OE^*, respectively. (B-E) *gasp1-1* mutant affects *PILS6^OE^* fluorescence and general growth under standard conditions of light and temperature. Confocal images (B) and quantification of signal intensity (C) show that *gasp1-1* reduces PILS6-GFP fluorescence. The white, dashed rectangle (B) shows the ROI used to quantify the signal intensity. Scans (D) and quantification (E) of root growth at 5 days after germination show that *gasp1-1* rescues the short root phenotype of *PILS6^OE^*. *n*=17, 15 (C) and 19 (E); ns, not significant, ***P<0.001, *t*-test and Mann–Whitney test (C) and one-way ANOVA and Tukey's multiple comparison test (E). Box plots extend from 25th to 75th percentile; horizontal lines represent median; whiskers represent minimum to maximum values. Scale bars: 100 μm (B); 0.5 cm (D).

Moderately high temperature induces PILS6 protein turnover, which consequently mediates auxin-dependent root thermomorphogenesis (Feraru et al., 2019; Fonseca de Lima et al., 2021), indicating that post-translational mechanisms define PILS activity and thereby plant adaptation. To further address these uncharted aspects of plant development, we performed a forward genetic screen, using a constitutive PILS6 expression line fused to GFP (*p35*::*PILS6-GFP*; hereafter named *PILS6^OE^*), and screened the progeny of about 5000 M1 ethyl methanesulfonate (EMS)-mutagenized *PILS6^OE^* seeds (Fig. 1A). We germinated the *PILS6^OE^* seedlings under standard growth conditions (21°C) for 4 days and, subsequently, shifted the plates for 24 h to 29°C. Afterwards, we evaluated the temperature-sensitive PILS6-GFP fluorescence intensity, using an epifluorescence microscope. After re-screening, we identified 21 mutants that showed either reduced (8) or enhanced (13) PILS6-GFP fluorescence intensity under these conditions. We accordingly named these mutants *gloomy and shiny pils* (*gasp*) (Fig. 1A).

### *gasp1* is a suppressor of PILS6

Among the eight mutants having reduced PILS6-GFP fluorescence signal intensity, we identified the *gasp1-1;PILS6^OE^* mutant that showed very weak PILS6-GFP fluorescence signal after 24 h exposure to 29°C (Fig. S1). Notably, when grown under standard temperature of 21°C, *gasp1-1* mutation caused a dramatic (85%) reduction of PILS6-GFP fluorescence intensity when compared with wild-type backgrounds (Fig. 1B,C; Fig. S1). This finding indicates that the *gasp1-1* mutation affects PILS6-GFP protein abundance independently of moderately high temperature. In accordance with its negative effect on the fluorescence intensity of PILS6-GFP, *gasp1-1* mutation alleviated the short root phenotype of *PILS6^OE^* by 15% (Fig. 1D,E). Therefore, we identified *gasp1-1* mutant as a suppressor of PILS6 under standard growth conditions.

### *GASP1* encodes a RING/U-box superfamily gene

**Fig. 2.**
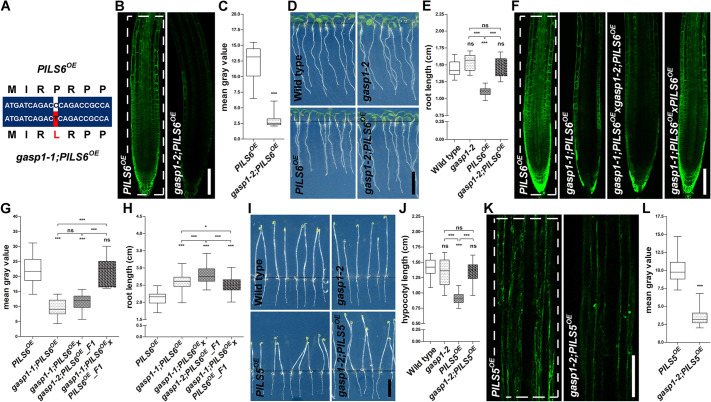
***gasp1* is defective in a RING/U-box superfamily gene.** (A) Alignment of a short nucleotide sequence from wild-type (top) and mutated (bottom) *GASP* gene. The mutated SNP and the changed amino acid are depicted in red. (B-E) The t-DNA insertion *gasp1-2* allele mimics the *gasp1-1* EMS mutant. Confocal images (B), scans of light-grown seedlings at 5 days after germination (D) and the respective quantifications (C,E) show that *gasp1-2* allele causes dramatic reduction of PILS6-GFP fluorescence in roots (B,C) and rescues the short root growth (D,E) of *PILS6^OE^*. *n*=15 (C) and 15-19 (E); ns, not significant; ***P<0.001, *t*-test and Mann–Whitney test (C); ***P<0.001, one-way ANOVA and Tukey's multiple comparison test (E). (F-H) Complementation test showing that *gasp* mutants are allelic. Confocal images (F) and quantifications of signal intensity (G) and root length (H) of the F1 crosses between *gasp1-1* and *gasp1-2* alleles in the *PILS6^OE^* backgrounds and the respective controls show that F1 *gasp1-1;PILS6^OE^*×*gasp1-2;PILS6^OE^* causes PILS6-GFP reduction in roots (F,G) and rescues the root growth defects of *PILS6^OE^* (H). *n*=10-12 (G) and 67-84 (H); ns, not significant, *P<0.05, ***P<0.001, one-way ANOVA and Tukey's multiple comparison test (G,H). (I-L) *gasp1-2* affects *PILS5^OE^* hypocotyl phenotype and PILS5-GFP fluorescence. Scans (I), confocal images (K) and quantifications of hypocotyl length (J) and signal intensity (L) show that *gasp1-2* mutant rescues the phenotype (I,J) and reduces PILS5-GFP signal intensity (K,L) in the dark-grown hypocotyls of *PILS5^OE^*. *n*=20-22 (J) and 15, 16 (L); ns, not significant; ***P<0.001, one-way ANOVA and Tukey's multiple comparison test (J); ***P<0.001, *t*-test and Mann–Whitney test (L). The white, dashed rectangles show the ROIs used to quantify the signal intensity. Box plots extend from 25th to 75th percentile; horizontal lines represent median; whiskers represent minimum to maximum values. Scale bars: 100 μm (B,F,K); 0.5 cm (D,I).

To identify the causal *GASP1* gene, we established a pool of *gasp1-1;PILS6^OE^* individuals isolated from an F2 backcross (*gasp1-1;PILS6^OE^* crossed to *PILS6^OE^*). Accordingly, we re-sequenced the genome of this pooled mutant population as well as a pool of non-mutagenized *PILS6^OE^* control seedlings using the Illumina and DNBseq^TM^ platform. By comparing the sequencing results of the two samples, we identified a single nucleotide polymorphism (SNP) in the uncharacterized protein-coding gene AT3G05545, which belongs to the RING/U-box superfamily protein (Kraft et al., 2005; Stone et al., 2005). The *gasp1-1* mutation causes a C-to-T mutation, resulting in a proline (P)-to-leucine (L) amino acid substitution at the position 274 (Fig. 2A; Fig. S2A). P274L is not in a conserved region of *GASP1*, but a leucine substitution of a proline residue may have dramatic structural and functional consequences (Vilsen et al., 1989; Molnar et al., 2016).

To confirm that *gasp1-1* mutation in AT3G05545 gene is indeed responsible for the suppression of *PILS6^OE^*, we isolated a second mutant allele (SALK_091345; hereafter called *gasp1-2*) from the Salk collection of t-DNA insertion lines (Alonso et al., 2003) (Fig. S2A). *GASP1* transcripts were not detectable in the *gasp1-2* allele, indicating a full knockout of *GASP1* (Fig. S2B). When crossed to *PILS6^OE^* (*gasp1-2;PILS6^OE^*), *gasp1-2* reduced PILS6-GFP fluorescence intensity and, consequently, rescued total root length (Fig. 2B-E). Next, we crossed *gasp1-1;PILS6^OE^* to the *gasp1-2;PILS6^OE^* allele as well as to the *PILS6^OE^* control line. In contrast to the control cross, the allelic test between *gasp1-1* and *gasp1-2* showed that the PILS6-GFP intensity and *PILS6^OE^* phenotypes remained suppressed in the F1 generation (Fig. 2F-H; Fig. S2C). Altogether, we concluded that defects in the *GASP1* are responsible for the phenotypes observed in the *gasp1-1;PILS6^OE^*.

### *GASP1* defines PILS5 and PILS6 protein abundance

To assess the specificity of GASP1, we crossed *gasp1-2* to the PILS5 overexpression line (*p35S::PILS5-GFP; PILS5^OE^*). Similar to *PILS6^OE^*, *PILS5^OE^*-induced reduction in main root growth was also suppressed in *gasp1-2;PILS5^OE^* (Fig. S2D,E). *PILS5^OE^* also represses dark-grown hypocotyl growth (Barbez et al., 2012; Beziat et al., 2017), which was as well alleviated by the *gasp1-2* mutation (Fig. 2I,J). In agreement, the PILS5-GFP signal intensity was strongly reduced in *gasp1-2;PILS5^OE^* dark-grown hypocotyls (Fig. 2K,L), showing that GASP1 affects at least two PILS proteins, in distinct tissues and growth conditions.

**Fig. 3.**
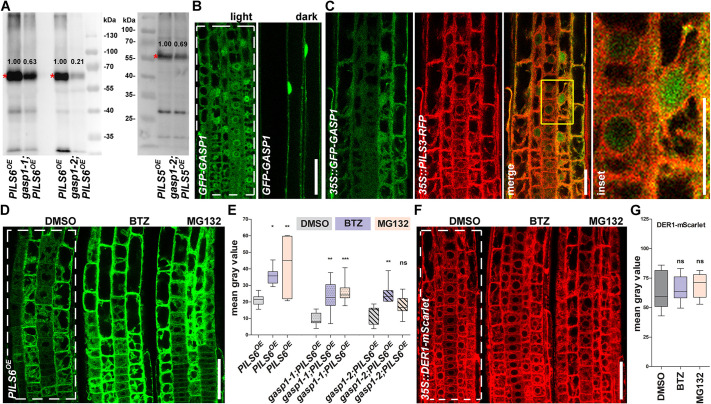
**GASP1 is an indirect regulator of PILS5 and PILS6 protein abundance.** (A) Western blots showing detection of PILS6-GFP (in light-grown seedlings, left) and PILS5-GFP (in dark-grown hypocotyls, right). Note the decreased abundance of both PILS5- and PILS6-GFP in the *gasp1* mutants. Red asterisks show PILS5- and PILS6-GFP bands. The values written above the GFP bands represent the intensities that were normalized to the tubulin (left) or Coomassie (right) bands presented in Fig. S3A or S3B, respectively. (B,C) *35S::GFP-GASP1* localizes to the cytosol and nucleus. GFP-GASP1 localization is shown in the roots (left) and hypocotyls (right) of light- and dark-grown seedlings from a homozygous F3 generation, respectively (B). GFP-GASP1 does not colocalize with the ER marker PILS3-RFP in an F1 cross (C). The yellow rectangle shows the region that is magnified in the inset. (D,E) Proteasome inhibitors stabilize PILS6-GFP independently of GASP1. Confocal images (D) and quantification of signal intensity (E) show that a short treatment (3 h) with the proteasome inhibitors BTZ (50 μM) or MG132 (50 μM) stabilizes PILS6-GFP in wild type (D,E) and in *gasp1* mutants (E). *n*=7-9; ns, not significant, *P<0.05, **P<0.01, ***P<0.001, one-way ANOVA and Tukey's multiple comparison test (E). (F,G) Proteasome inhibitors do not affect DER1-mScarlet. Confocal images (F) and quantification of signal intensity (G) show that a 3 h treatment with the proteasome inhibitors BTZ (50 μM) or MG132 (50 µM) does not affect DER1-mScarlet fluorescence intensity. *n*=11-13; ns, not significant, one-way ANOVA and Tukey's multiple comparison test (G). The white dashed rectangles show the ROIs used to quantify the signal intensity. Box plots extend from 25th to 75th percentile; horizontal lines represent median; whiskers represent minimum to maximum values. Scale bars: 50 μm (B,D,F); 25 μm (C).

To directly address whether GASP1 indeed affects PILS5 and PILS6 protein abundance, we subsequently used quantitative western blots. In accordance with the reduced PILS5/6-GFP fluorescence intensity, *gasp1* mutants displayed reduced PILS5 and PILS6 protein levels in the dark-grown hypocotyls and light-grown seedlings, respectively (Fig. 3A; Fig. S3A,B). We conclude that GASP1 defines the abundance of PILS proteins, such as PILS5 and PILS6.

GASP1 belongs to the RING/U-box superfamily and plays a role as an E3 ubiquitin ligase (Kraft et al., 2005; Stone et al., 2005). RING E3 ubiquitin ligases typically mediate the ubiquitylation of target proteins, where K48-linked ubiquitylation recruits these targets for degradation via the 26S proteasome (Joazeiro and Weissman, 2000; Smalle and Vierstra, 2004; Vierstra, 2009). To investigate whether GASP1 could directly modulate PILS protein abundance at the ER membrane, we generated a transgenic line overexpressing GFP-GASP1 fusion. *35S::GFP-GASP1* lines displayed weak but ubiquitous signal in the root (Fig. S3C). In agreement with its predicted localization (https://suba.live/suba-app/factsheet.html?id=AT3G05545; Hooper et al., 2017), GFP-GASP1 was detectable in the nucleus, but also showed cytosolic localization (Fig. 3B). Although GFP-GASP1 appeared to be enriched in the perinuclear regions of light-grown seedlings, we did not detect pronounced association with the ER and, accordingly, GFP-GASP1 did not show co-localization with PILS3-RFP (Fig. 3B,C). In addition, GASP1 did not interact with PILS3 or PILS5 proteins in a yeast mating-based split-ubiquitin system (Fig. S3D). Even though we cannot fully rule out a direct interaction *in planta*, we assume that the putative E3 ubiquitin ligase GASP1 indirectly affects the protein abundance of PILS5 and PILS6. Hence, we propose that GASP1 may define the ubiquitylation and subsequent proteasomal degradation of cytosolic and/or nuclear proteins that are upstream regulators of PILS5 and PILS6 proteins. To test whether the degradation of the PILS proteins is affected by the disturbance of the ubiquitin-26S proteasome pathway, we subsequently used MG132 and Bortezomib (BTZ) to pharmacologically interfere with the proteasome function in *Arabidopsis* (Marshall et al., 2015; Yu et al., 2015; Wang et al., 2016). Seedlings of *PILS6^OE^* were treated for 3 h with MG132 or BTZ, which caused a significant increase of PILS6-GFP fluorescence in roots when compared with the DMSO-treated control seedlings (Fig. 3D,E), indicating that PILS6 protein abundance is regulated in a 26S proteasome-sensitive manner. Importantly, another ER membrane-localized protein, the RING E3 ligase DER1 (AT4G29330; Kirst et al., 2005), a component of the ERAD pathway, translationally fused to mScarlet (*35S::DER1-mScarlet*) is not affected by proteasome inhibition, indicating some specificity of this effect (Fig. 3F,G). The pharmacological inhibition of the 26S proteasome also increased the PILS6-GFP fluorescence in *gasp1-1* and *gasp1-2* mutants (Fig. 3E; Fig. S3E-G), indicating that other molecular components contribute to the effect of MG132 and BTZ on PILS6 abundance. Collectively, our data shows that, although the 26S proteasome activity is required for PILS6 degradation, PILS protein abundance is not directly controlled by the E3 ubiquitin ligase GASP1.

### Auxin signaling modulates PILS6 protein abundance

**Fig. 4.**
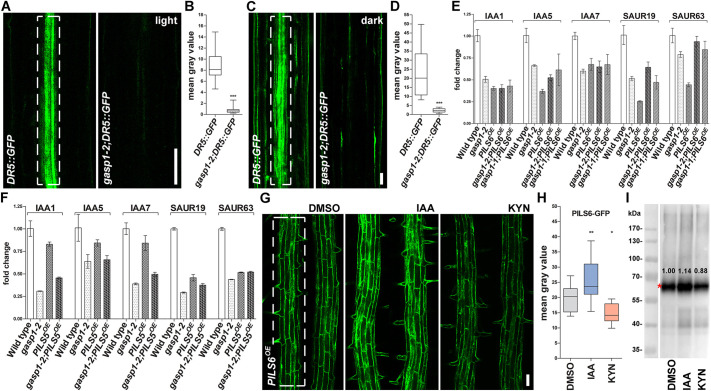
**Auxin signaling affects PILS6 protein abundance.** (A-F) Auxin signaling is reduced in the *gasp1* mutants. Confocal images (A,C) and quantifications of signal intensity (B,D) show that *gasp1-2* mutation negatively affects *DR5::GFP* signal in the upper root (differentiation zone is presented) of light-grown (A,B) and hypocotyls of dark-grown (C,D) seedlings. qPCR analysis showing the expression of some IAA and SAUR genes in the entire seedlings grown in the light (E) and hypocotyls of seedlings grown in the dark (F). Note the reduced expression of the auxin-responsive genes in all transgenic lines. *n*=16, 17 (B) and 9, 10 (D); ***P<0.001, *t*-test and Mann–Whitney test (B,D). (G-I) Auxin signaling modulates PILS6 protein abundance. Confocal images (G), quantification of signal intensity (H) and immunoblot with anti-GFP (I) show that 24 h treatment with either IAA (100 nM) or KYN (1uM) increases or reduces PILS6-GFP abundance in roots of light-grown *PILS6^OE^* seedlings. Red asterisk (I) marks PILS6-GFP bands. The values written above the GFP bands represent the intensities that were normalized to the tubulin bands presented in Fig. S4C. The white, dashed rectangles show the ROIs used to quantify the signal intensity. *n*=17; *P<0.05, **P<0.01, one-way ANOVA and Tukey's multiple comparison test (H). Box plots (B,D,H) extend from 25th to 75th percentile; horizontal lines represent median; whiskers represent minimum to maximum values. Data are mean±s.e.m. (E,F). Scale bars: 50 μm (A,C,G).

We next addressed whether the reduced PILS5 and PILS6 protein abundance correlates with the expected increased nuclear auxin signaling output in *gasp1* (Barbez et al., 2012; Beziat et al., 2017; Feraru et al., 2019; Sun et al., 2020). To visualize the auxin signaling output in *gasp1-2*, we crossed *gasp1-2* with the auxin response marker *DR5::GFP*. Although *DR5::GFP* signal intensity was not distinguishable in the root tip of *gasp1-2* mutant and wild type (Fig. S4A,B) we, unexpectedly, observed reduced *DR5::GFP* signal in the upper vascular tissues of light-grown roots as well as dark-grown hypocotyls of *gasp1-2* mutant (Fig. 4A-D). In agreement, auxin responsive genes, such as *IAA1*, *IAA5*, *IAA7*, *SAUR19* and *SAUR63*, showed reduced expression in the light-grown seedlings and in the dark-grown hypocotyls of *gasp1* mutants (Fig. 4E,F). This finding suggests that *GASP1* is required to maintain auxin signaling output in roots and shoots, which is independent from its effect on PILS5 and PILS6 abundance (Fig. 4E,F). The overexpression of PILS proteins also limits nuclear auxin signaling, but the repression of auxin signaling output was not additive in *gasp1-1;PILS6^OE^*, *gasp1-2;PILS6^OE^* and *gasp1-2;PILS5^OE^* (Fig. 4E,F), suggesting that the effect on PILS abundance balances the auxin response. This finding hints at a molecular mechanism in which the PILS abundance could relate to a homeostatic feedback mechanism on auxin signaling output.

This prompted us to address whether the diminished auxin signaling output observed in *gasp1* mutants could reflect an auxin impact on PILS proteins abundance. To test this, we used L-Kynurenin (KYN) to pharmacologically interfere with auxin biosynthesis (He et al., 2011). KYN applications indeed phenocopied *gasp1* mutants and decreased the PILS6-GFP fluorescence as well as protein abundance (Fig. 4G-I; Fig. S4C). Conversely, 100 nM IAA treatment increased both PILS6-GFP fluorescence and protein abundance (Fig. 4G-I; Fig. S4C). These experiments suggest that high and low auxin signaling outputs increase and decrease PILS6 abundance, respectively. In contrast, the ER membrane marker DER1-mScarlet did not show any response to the treatments with either KYN or IAA (Fig. S4D,E), suggesting certain specificity of this auxin response. This data proposes that auxin exerts a homeostatic feedback on its own signaling rate by controlling the abundance of PILS intracellular auxin transporters.

## DISCUSSION

Our forward genetic screen performed to identify regulators of the PILS6 response to moderately high temperature yielded 21 *gasp* mutants that either decreased (*gloomy*) or increased (*shiny*) PILS6 protein abundance. In this study, we investigated *gasp1-1* that repressed PILS6-GFP under standard temperature and found that *GASP1* may function as a modulator of auxin signaling rates. *GASP1* encodes an uncharacterized E3 ubiquitin ligase, from the H-type, that belongs to the RING/U-box superfamily protein, which supposedly mediates substrate specific ubiquitylation (Kraft et al., 2005; Stone et al., 2005). The *gasp1* mutants display severe reduction in auxin signaling output but did not obviously increase phenotypic trait variations, being largely indistinguishable from wild-type seedlings. It is therefore conceivable that homeostatic auxin responses may balance the molecular responses in *gasp1*.

The biological role of *GASP1* remains largely unknown, but we show here that the severely reduced auxin signaling output in *gasp1* mutants is in part compensated by enhanced turnover of at least PILS5 and PILS6 proteins. Our data show that GASP1 does not directly interact with PILS proteins, such as PILS3 or PILS5, heterologously expressed in yeast. Considering that E3 ligases are typically negative regulators of their clients, the reduced PILS5 and PILS6 abundance in *gasp1* mutants also questions a direct impact of the GASP1 on the PILS proteins. We propose that the GASP1 impact on auxin signaling output indirectly affects PILS5 and PILS6 turnover. We, accordingly, show that sub- and supra-optimal levels of auxin decrease and increase PILS6 abundance at the ER, respectively. It remains to be seen how precisely auxin levels determine the turnover of PILS proteins. Such a response could involve the canonical TIR1/AFB auxin receptors and downstream signaling events, altering the yet-to-be-defined PILS degradation mechanisms. Alternatively, auxin availability may structurally affect PILS proteins, which could alter their interaction with the degradation machinery.

PILS proteins define the nuclear abundance and signaling of auxin, which appears to be highly responsive to internal and environmental signal perturbations (Beziat et al., 2017; Feraru et al., 2019; Sun et al., 2020). Here, we propose a working model where an auxin impact on PILS abundance provides homeostatic feedback (Fig. S4F), enabling auxin signaling output to maintain its own cellular homeostasis. Altogether, we envision that a PILS-dependent feedback mechanism provides robustness to plant growth and development.

## MATERIALS AND METHODS

### Plant material

*Arabidopis thaliana* ecotype Col-0 (wild-type), *p35S::PILS5-GFP* (*PILS5^OE^*; AT2G17500; Barbez et al., 2012), *p35S::PILS6-GFP* (*PILS6^OE^*; AT5G01990; Barbez et al., 2012) and *pDR5rev::GFP* (Benkova et al., 2003) have been previously described. *p35S::GFP-GASP1* (AT3G05545), *p35S::PILS3-RFP* (AT1G76520) and *35S::DER1-mScarlet* (AT4G29330) were generated in this study. *gasp1-2* (SALK_091345) was obtained from NASC (Alonso et al., 2003); *gasp1-1* was identified in this study.

### Growth conditions

Seeds were sterilized in 70% ethanol (1-2 min sterilization in paper bags, followed by 30 min drying) and plated, usually on one single line, uniformly spaced, in the upper part of Petri dishes containing 50 ml solidified Murashige and Skoog (MS) agar medium (0.8% agar, 0.5×MS and 1% sucrose, pH 5.9), then stratified for 3 days in the dark at 4°C and grown on vertically oriented plates in a plant cabinet equipped with above-placed cool-white fluorescent bulbs set at about 140 μmol/m^−2^s^−1^, long-day photoperiod (16 h light/8 h dark) at 21°C. This way of plating ensures that all seedlings in the plate are exposed to the same light intensity and humidity, which results in low variability. For moderately high temperature (HT)-related experiments, we used the growth conditions described in Feraru et al. (2019). Seedlings were grown on plates (in pairs), for 4 days at 21°C (standard temperature) and subsequently shifted for 24 h to a cabinet displaying similar settings, excepting the temperature was 29°C (moderately high temperature). The control plates remained in the cabinet equipped with standard conditions.

### EMS mutagenesis, forward genetic screen and sequencing

Approximately 10,000 seeds of *35S::PILS6-GFP (PILS6^OE^)* were soaked (gently shaking) for 10 h in 0.1 M phosphate buffer, pH 7, containing 0.3% (v/v) EMS. Before mutagenesis, the seeds were soaked for 5 min in water containing 0.05% Triton X-100, then rinsed three times with water. After mutagenesis, the seeds were rinsed seven times with water, then dispersed as desired in 0.1% agarose and transferred to soil by pipetting. From ∼5400 mutagenized plants (M1), we harvested 360 pools (each pool containing about 15 M1), and screened more than 80,000 M2 seedlings under an Olympus stereomicroscope for individual seedlings with weaker or stronger fluorescence than *PILS6^OE^* control. The seedlings having different fluorescence intensity than the control were picked up, propagated, and confirmed in the next generation as *gasp* mutants.

For sequencing of *gasp1-1*, we crossed *gasp1-1;PILS6^OE^* to *PILS6^OE^* and selected in F2 the individuals showing the *gasp1-1;PILS6^OE^* phenotype. A pool of seedlings weighing 100 mg was used to extract genomic DNA using the DNeasy Plant Mini Kit (Qiagen), according to the manufacturer's instructions. A sample of more than 1.5 µg (>13 ng/µl sample concentration) was sent for sequencing at BGI Tech Solutions, along with a similar sample containing the *PILS6^OE^* control. The samples were sequenced using the DNBseq platform and the standard bioinformatics analysis was performed by BGI Tech Solutions, which identified the different SNPs between each sample and *Arabidopsis thaliana* genome from the TAIR database, followed by SNP calling, annotation and statistics. To identify the *gasp1-1* mutation, we compared the list of SNPs identified in the *gasp1-1;PILS6^OE^* with the list of SNPs identified in the *PILS6^OE^* sample. After elimination of common SNPs between the two samples and of those heterozygous and synonymous SNPs, we identified one single typical EMS mutation.

### Quantification of phenotypes

For root and hypocotyl length measurements, seedlings were grown on vertically oriented plates in the light (root) or dark (hypocotyl). Plates were scanned with an Epson Perfection V700 scanner and the length was measured using ImageJ 1.41 software (http://rsb.info.nih.gov/ij/).

### Confocal imaging and quantification

A Leica TCS SP5 confocal microscope was used for fluorescence imaging. Unless otherwise stated, 5-day-old seedlings were used. When treated before imaging, the seedlings were either submerged in MS liquid medium (MG132, BTZ) or transferred on plates (IAA, KYN) containing the desired concentration of the drug or similar amount of solvent and kept in the plant cabinet for the duration specified in the text or figure legend. The mean gray value of the fluorescence intensity was quantified in a defined rectangle region of interest (ROI), marked on the images, using the ‘Quantify’ tool of Leica software (LAS AF Lite).

### Cloning

To generate *p35S::GFP-GASP1*, the GASP genomic fragment was cloned into the pDONR221 using the primers B1_GASP_FP and B2_GASP^STOP^_RP, listed in Table S1. The resulting entry clone was subsequently transferred to the gateway-compatible destination vector pK7WGF2 (Karimi et al., 2002). Transformed lines were selected on 100 mg/l kanamycin in F1 and 25 mg/l kanamycin in F2. For the split ubiquitin assay, we amplified the PILS3, PILS5 and GASP1 coding sequence without stop codon using PILS3_FP and PILS3^NOSTOP^_RP, PILS5_FP and PILS5^NOSTOP^_RP, B1_GASP_FP and B2_GASP^NOSTOP^_RP listed in Table S1. The fragments were firstly cloned into the pDONR221. Subsequently, we recombined the baits (PILS3 and PILS5) and prey (GASP1) into pMetYC-DEST (Grefen et al., 2007) and PNX35-DEST (Grefen and Blatt, 2012), respectively. We used Gibson Assembly (New England Biolabs) to generate *35S::DER1-mScarlet*. The coding sequence of DER1 (DER1_FP and DER1_RP), the 35S promoter (35S_FP and 35S_RP) and mScarlet-i tag (mScarlet_FP and mScarlet_RP) were amplified by PCR using Q5 High-Fidelity DNA Polymerase (New England Biolabs). The fragments were then cloned into a linearized (EcoRV-HF-NEB) pPLV03 vector using Gibson Assembly. The transformed lines were selected on 15 mg/l phosphinothricin (Basta). The *35S::PILS3-RFP* plasmid generated previously (Barbez et al., 2012) was transformed into Col-0 plants and the transformed lines were selected on 20 mg/l hygromycin.

### Split ubiquitin

For the split ubiquitin assay, the yeast strains THY.AP4 and THY.AP5 were transformed with bait and prey constructs, respectively, using a modified protocol from Gietz and Woods (2002). Approximately 100 µl of fresh yeast were scraped from YPD plates, resuspended in 200 µl sterile H_2_O, centrifuged for 5 min at 2000 ***g*** and the supernatant removed. The yeast was afterwards resuspended in 200 µl yeast transformation buffer (40% PEG 3350, 200 mM LiAc, 100 mM DTT), added 10 µl single stranded carrier DNA and 1 µg of plasmid DNA, and mixed by pipetting up and down. We incubated the yeast for 15 min at 30°C and for 45 min at 45°C, subsequently plated on Synthetic Defined (SD) medium, and incubated for 4 days at 28°C. A pool of transformed colonies was mated as described in Grefen et al. (2007). The selected diploid colonies were afterwards incubated on plates contacting selective medium (SD-Trp, -Leu, -Ade, -His, -Ura) at 21°C, under light (16 h light/8 h dark) and dark (plates were wrapped in aluminum foil to mimic dark) conditions. Growth was recorded up to 9 days after plating.

### Sequencing and genotyping

To identify *gasp1-1*, we amplified the genomic sequence with B1_GASP1_FP and B2_GASP1^STOP^_RP and sequenced the sequence around the mutation using the primer GASP1_FP6 listed in Table S1. To genotype *gasp1-2*, we used a combination of *gasp1*-*2* and t-DNA insertion-specific primers listed in Table S1.

### RT-qPCR analysis

RT-qPCR analysis was performed as described in Feraru et al. (2019). We used the InnuPREP Plant RNA Kit (Analytic Jena) to extract total RNA according to the manufacturer's recommendation. The RNA samples were treated with InnuPREP DNase I (Analytic Jena). To synthesize cDNA, 1 μg of RNA and the iSCRIPT cDNA Synthesis Kit (Bio-Rad) were used. RT-qPCR was carried out in a C1000 Touch Thermal Cycler equipped with the CFX96 Touch Real-Time PCR Detection System (Bio-Rad) and using the Takyon qPCR Kit for SYBER assay (Eurogentec), according to the manufacturer's recommendation. Gene expression was normalized to the expression of reference gene *ACTIN 2*, which showed similar expression among genotypes. We used RealTimeDesign qPCR Assay Design Software from Biosearch Technologies to design the qPCR primers or we used primers already published (Inoue et al., 2016; Schlereth et al., 2010). The primers used for qPCRs are listed in Table S1.

### Western blots

We used 5-day-old dark-grown hypocotyls for the experiment related to *PILS5^OE^* and 6-day-old total seedlings for the experiments related to *PILS6^OE^*. For IAA and KYN treatments, the seedlings were grown for 5 days on nylon mesh on MS plates, then transferred with the underlying mesh to the plates supplemented with IAA, KYN or similar amount of DMSO solvent, and harvested after 24 h. Protein extraction was performed as described in Moulinier-Anzola et al. (2020). Each sample contained 20 mg seedlings. The frozen plant material was ground and then extracted in 150 μl buffer [65 mM Tris (pH 6.8), 8 M urea, 10% glycerin, 2% SDS, 5% β-mercaptoethanol and 0.25% bromophenol blue]. The samples were then heated at 65°C for 5 min and spun down before loading. Anti-GFP (Roche, 11814460001, 1:1000), monoclonal anti-α-Tubulin (Sigma-Aldrich, T6074, 1:100,000) and goat anti-mouse IgG (Jackson ImmunoResearch, 115-035-164, 1:40,000) antibodies were used for detection of *PILS5^OE^*, *PILS6^OE^* and Tubulin.

### Statistics

We used Excel to organize data and GraphPad Prism to generate graphs and perform statistical analysis. The experiments were performed at least three times or in three replicates, but only the data from one representative experiment is shown.

## Supplementary Material

Supplementary information

Reviewer comments
