# Supplementary material for: PILS proteins provide a homeostatic feedback on auxin signaling output
Source: Development. 2022 Jul 12;149(13):dev200929. doi: 10.1242/dev.200929 (PMC9340555; doi:10.1242/dev.200929)
Supplement: Supplementary information [file develop-149-200929-s1.pdf]

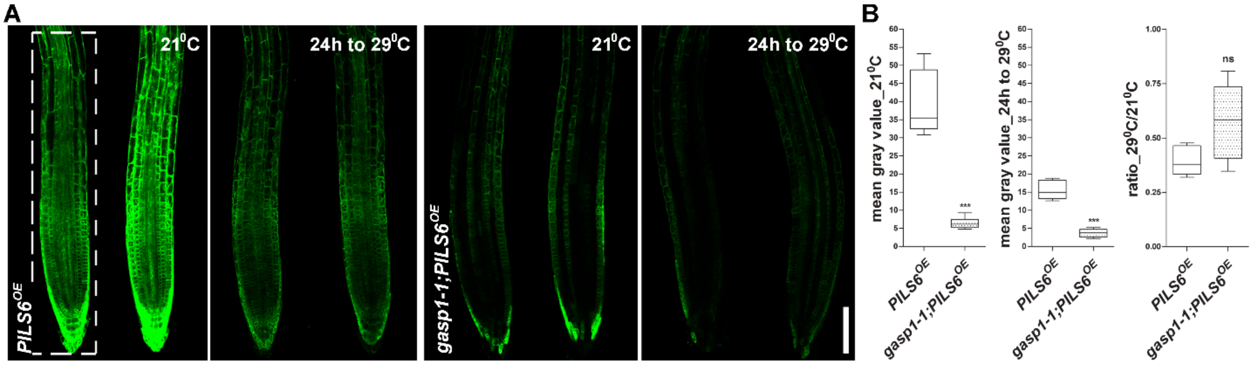

Fig. S1. ***gasp1-1*** **regulates PILS6 independently of moderately high temperature**

A,B.*gasp1-1* mutation affects *PILS6*^{OE} already under standard growth conditions. Confocal images (A) and quantification of signal intensity (B) show that PILS6-GFP fluorescence is already weaker in the *PILS6*^{OE} seedlings grown under 21 ^{O}C and is further reduced, similarly to the control seedlings, after 24 h exposure to 29°C. n = 8; ns = not significant, ***P = 0.0007, t-test and Mann-Whitney test (B). Scale bar, 100 µm (A).

The white, dashed rectangle shows the ROI used to quantify the signal intensity.

Development • Supplementary information

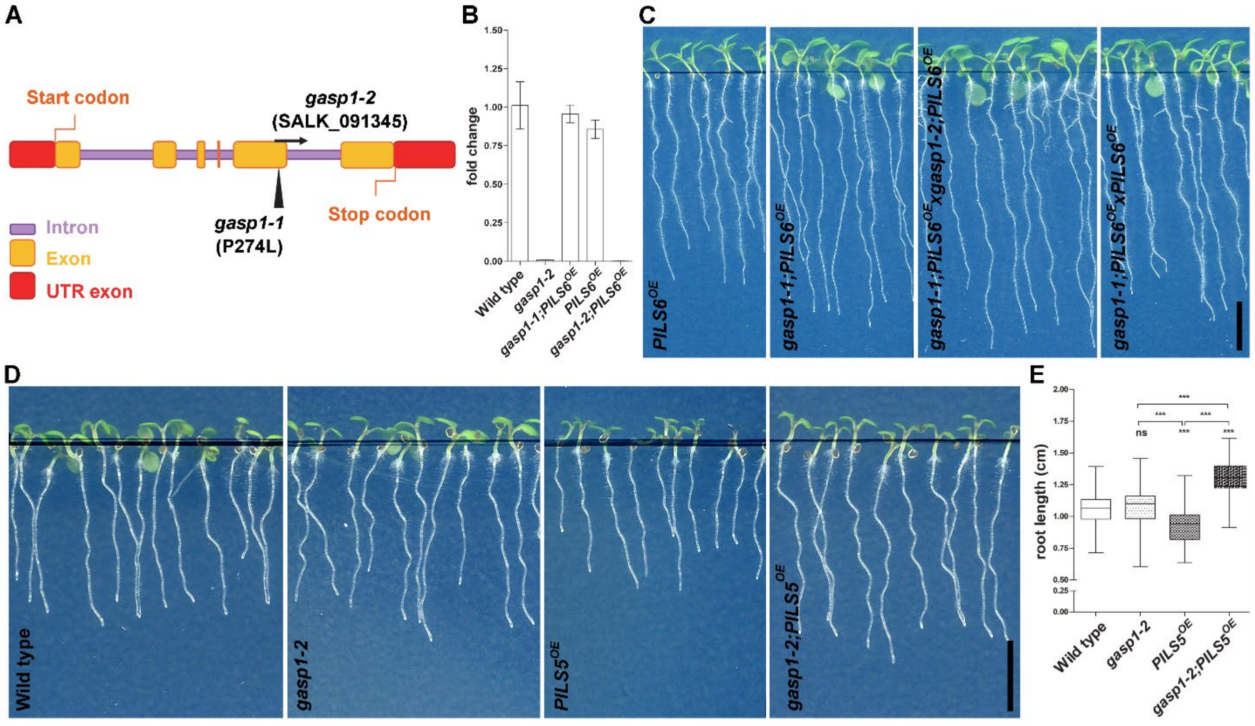

Fig. S2. *GASP1* encodes a RING/U-box superfamily gene

A.Schematic representation of *GASP1* gene, according to PLAZA 5.0 (https://bioinformatics.psb.ugent.be/plaza/versions/plaza_v5_dicots/). Black arrowhead and arrow show the approximate positions of *gasp1-1* SNP and *gasp1-2* t-DNA insertion, respectively.

B.qPCR showing *GASP1* transcript. *GASP1* transcript is absent in *gasp1-2* mutant and unchanged in *gasp1-1* mutant. Wild type and *PILS6*^{OE} were used as controls.

C.*gasp1* mutants are allelic. Scans of 7 DAG seedlings show that the F1 cross between *gasp1-1* and *gasp1-2* mutants in *PILS6*^{OE} background rescues the short root growth of *PILS6*^{OE}. Scale bar, 0.5 cm.

D,E.*gasp1-2* affects *PILS5*^{OE} root phenotype. Scans (D) and quantification (E) show that *gasp1-2* allele rescues root growth of 5 DAG light-grown *PILS5*^{OE} seedlings. n = 41-43; ns = not significant, ***P < 0.05, One-way ANOVA and Tukey's multiple comparison test (E). Scale bar, 0.5 cm (D).

Development • Supplementary information

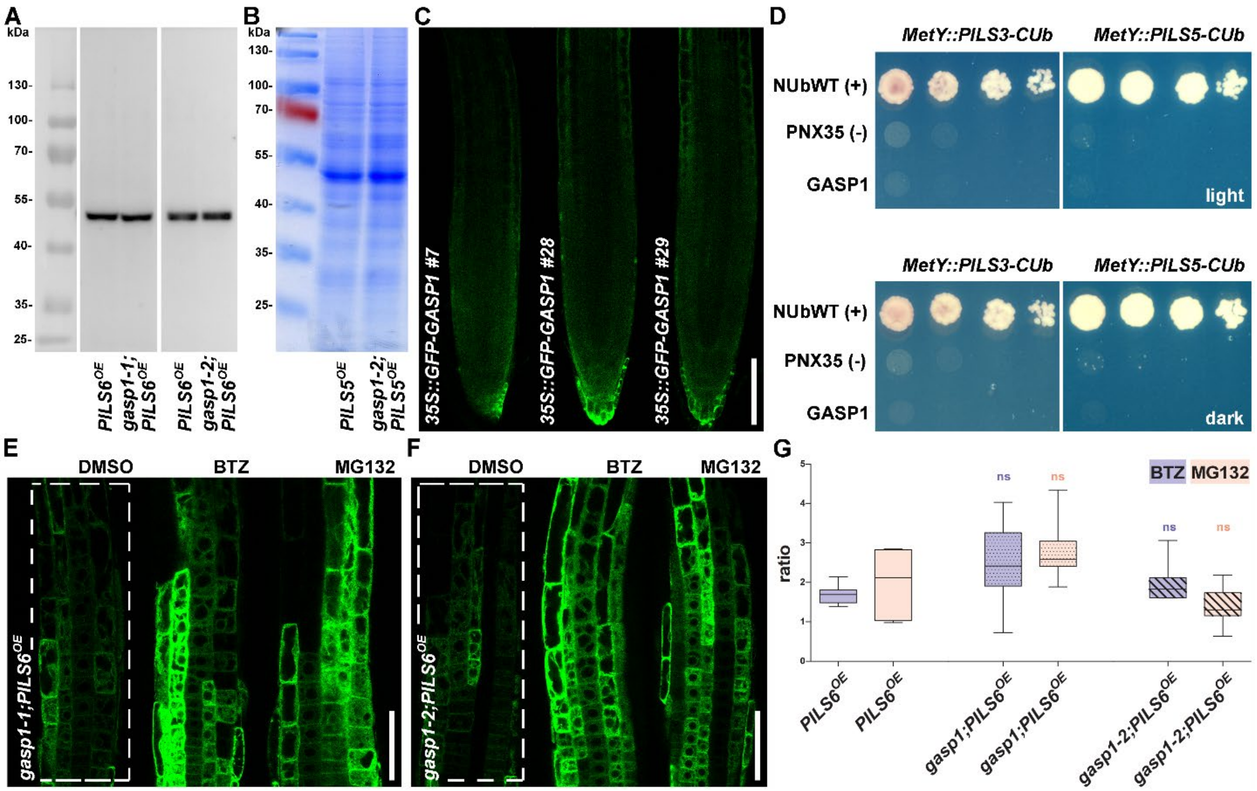

Fig. S3. GASP1 affects indirectly the proteasome-dependent PILS6 protein abundance

A,B.Controls for PILS5- and PILS6-GFP Western blots. Anti-Tubulin- (A) and Coomassie- (B) based normalizations were used for the Western blot analyses presented in Figure 3A.

C.*35S::GFP-GASP1* localization in roots. Three independent lines show weak but ubiquitous localization in 5 DAG light-grown seedlings. We used mainly line 28. Scale bar, 100 µm.

D.PILS3 and PILS5 proteins do not interact with GASP1. Neither PILS3 nor PILS5 interact with GASP1 in the light (upper image) or dark (lower image) in the yeast mating-based split-ubiquitin system. NUbWT was used as a positive control, PNX35 as a negative control.

E-G.Proteasome inhibitors stabilize PILS6-GFP independently of GASP1. Confocal images (E, F) and BTZ/DMSO and MG132/DMSO ratios of signal intensity (G) show that a short treatment (3 h) with theproteasome inhibitors Bortezomib (BTZ; [50 uM]) or MG132 [50 uM] stabilizes PILS6-GFP in WT and *gasp1* mutants. The ratios were calculated with the values from Figure 3E. ns = not significant, One-way ANOVA and Tukey's multiple comparison test (G). Scale bars, 50 µm (E, F).

The white, dashed rectangles show the ROIs used to quantify the signal intensity.

Development • Supplementary information

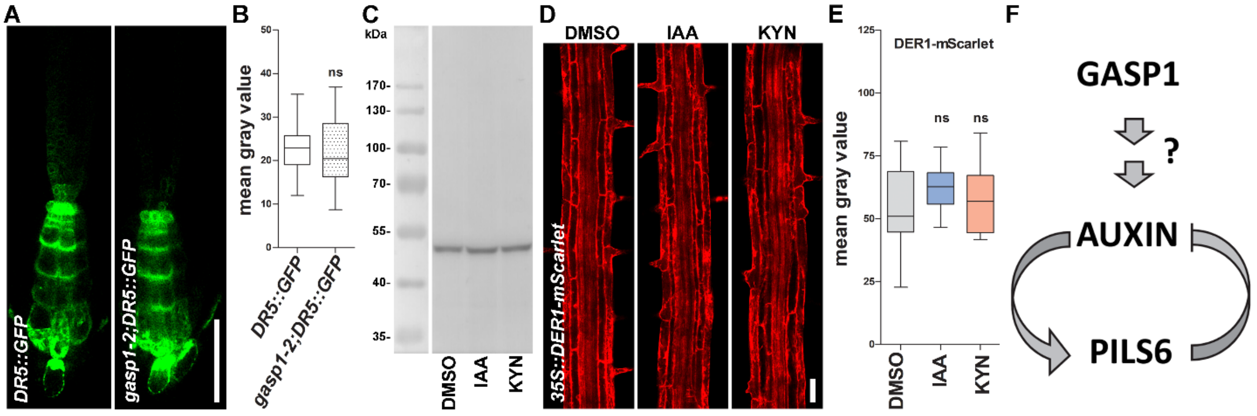

Fig. S4. Auxin feedback on PILS proteins

A,B.*DR5::GFP* signal intensity is not affected in the very root tip of *gasp1-2* seedlings. Confocal images (A) and quantification of signal intensity (B) show slightly but not significantly weaker *DR5::GFP* signal intensity in the root tip of *gasp1-2* mutant grown in the light for 5 DAG. n = 15, 16; ns = not significant, t-test and Mann-Whitney test (B). Scale bar, 50 µm (A).

C.Anti-Tubulin-based normalization was used for the Western blot analysis presented in Figure 4I.

D,E.Auxin signaling does not affect *35S::DER1-mScarlet*. Confocal images (D) and quantification of signal intensity (E) show that a 24 h treatment with either [100 nM] IAA or [1uM] KYN does not affect the fluorescence of DER1-mScarlet. n = 11; ns = not significant, One-way ANOVA and Tukey's multiple comparison test (E). Scale bar, 50 µm (D).

F.Working model illustrating our findings. GASP1 modulates (directly or not) auxin signaling output, which further influences PILS6 protein stability. In return, PILS6 activity represses the abundance of auxin for nuclear auxin signaling. This intracellular feedback regulation between auxin and PILS6 may allow an optimal auxin concentration for fine-tuning auxin-dependent plant responses.

Table S1. Primers used in this study.

Name	Sequence	Reference
Cloning (and PCR amplification for sequencing)		
B1_GASP1_FP	GGGGACAAGTTTGTACAAAAAAGCAGGCTTTATGGGTTTAGGCAATAAGGGT	This study
B2_GASP1^{STOP}_RP	GGGGACCACTTTGTACAAGAAAGCTGGGTTTAAGATGATCCTCCTCCGCC	This study
B2_GASP1^{NOSTOP}_RP	GGGGACCACTTTGTACAAGAAAGCTGGGTTAGATGATCCTCCTCCGCCA	This study
PILS3_FP	GATATCGAATTCCTGCAGCCCGGGGATGGTGAAGCTTTTGGAG	This study
PILS3^{NOSTOP}_RP	AAAGCTGGAGCTCCACCGCGGTGGCCTAAGCTACAAGCCACATG	This study
PILS5_FP	GATATCGAATTCCTGCAGCCCGGGGATGGGATTCTGGTCGTTG	This study
PILS5^{NOSTOP}_RP	AAAGCTGGAGCTCCACCGCGGTGGCTTAGACTAACAAGTGAAGGAAG	This study
DER1_FP	CTATTCTAGTCGAATGTCTTCTCCTGGCGAATTC	This study
DER1_RP	GCCCTTGCTCACGTCGGTGAGACGATATGATC	This study
35S_FP	GGTCGACGGTATCGATAAGCTTGATGACTAGAGCCAAGCTGATC	This study
35S_RP	AGGAGAAGACATTCGACTAGAATAGTAAATTGTAATGTTG	This study
mSarlet_FP	TCGTCTCACCGACGTGAGCAAGGGCGAGGCA	This study
mSarlet_RP	TAACCCATTCCAACTAGAATTCGATCATAGATGACACCGCGCGC	This study
Sequencing		
GASP1_FP6	CTTCAATTATGTTCCATCTCG	This study
Genotyping		
gasp1-2_FP	CCGAATTCAATGTCGAGGAT	This study
gasp1-2_RP	TAAACCTGTGGTATCACGAA	This study
Salk_LB_1-3	ATTTTGCCGATTTCGGAAC	SIGnAL
qPCR		
IAA1_FP	GTCAAAAACTCAGAATCATGAAAGGA	This study
IAA1 _RP	TGCCTCGACCAAAAGGTGTT	This study
IAA5 _FP	AGACTGTTCTTTCTCCGGTACGA	This study
IAA5 _RP	ACCGGCGAAAAAGAGTCAAG	This study
IAA7 _FP	TGAACGAGAGCAAGCTAATGAATC	This study
IAA7 _RP	AACGAGCATCCAGTCACCATCT	This study
SAUR19 _FP1	GGCTTAACGATCCCTTGTCCC	Inoue et al., 2016
SAUR19 _RP1	TTTACAATGAATAAGTCTATTTCTAACTGAAGGA	Inoue et al., 2016
SAUR63 _FP	CTGTTGTCCAGGAGCTATTGAAA	This study
SAUR63 _RP	GGCCGAATCGAATGGTAATGTG	This study
ACT2_FP	ATTCAGATGCCCAGAAGTCTTGTTC	Schlereth et al., 2010
ACT2_RP	GCAAGTGCTGTGATTTCTTTGCTCA	Schlereth et al., 2010
GASP1_FP	GGAGGCCCGCTAGAGGAAT	This study
GASP1_RP	CCCACCTGCCTGATCTGAAG	This study

Development • Supplementary information
