# Supplementary material for: PILS proteins provide a homeostatic feedback on auxin signaling output
Source: Development. 2022 Jul 12;149(13):dev200929. doi: 10.1242/dev.200929 (PMC9340555; doi:10.1242/dev.200929)
Supplement: Reviewer comments [file develop-149-200929_review_history.pdf]

PILS proteins provide a homeostatic feedback on auxin signaling output

Elena Feraru, Mugurel I. Feraru, Jeanette Moulinier-Anzola, Maximilian Schwihla, Jonathan Ferreira Da Silva Santos, Lin Sun, Sascha Waidmann, Barbara Korbei and Jürgen Kleine-Vehn

DOI: 10.1242/dev.200929

Editor: Yka Helariutta

Review timeline

Submission to Review Commons:	18 December 2021
Submission to Development:	9 May 2022
Accepted:	7 June 2022

Reviewer 1

Evidence, reproducibility and clarity

Summary: This manuscript identifies a RING/U-box protein, named GASP1, as a putative novel regulator of PILS5 and PILS6 abundance and auxin response in Arabidopsis seedlings. The novel data is high quality and of great interest to the auxin and protein ubiquitination community.

Major Comments:

• If GASP regulates (directly or indirectly) the degradation of PILS6 then one might expect increased levels of PILS6 in the absence of GASP. However, the opposite is observed (i.e. reduced levels of PILS6 in gasp1-1). In addition, the MG132 and BTZ treatments of PILS6OE lines indicate increased protein abundance. An alternative model might be that GASP-mediated ubiquitination of PILS6 is required for protein activation and/or subcellular localization, which has been reported for other eukaryotic ubiquitinated substrates. A working model for GASP and PILS would help synthesize the authors findings; perhaps this can be added as a final supplemental figure?

• The light regulated dynamics of GASP1 localization (Figure 3) is quite intriguing! In addition, the data shown in Figure 3B and 3C suggests that GASP and PILS6 could interact at the plasma membrane, perhaps via a complex.

• Can the authors speculate on the effects of the gasp1-1 allele and how the P274L mutation may impact GASP function? It is interesting that this allele and the null allele (gasp1-2) have the same genetic effect, but also a bit puzzling.

Minor Comments:

• Previous work has described and demonstrated that PILS proteins are auxin "transporters", which is a bona fide protein functional category. Use of the term auxin "facilitator" (lines 22, 39 and 145) may be confusing for readers as it is not technically a protein functional category. The PILS proteins may be described as facilitating transport, but I would caution against using this term as a noun.

• In the Cloning section of the methods, line 226, please add the concentration of kanamycin used for transgenic selection.

• Figure 1A legend: suggest modifying the sentence "Overall, the gloomy mutants were longer and the shiny mutants were shorter" (lines 339-340) to "Overall, the gloomy mutant roots were longer and the shiny mutant roots were shorter".

• Figure 2I-L legend: suggest modifying the title "GASP1 is not specific to PILS6" (line 363) to "GASP1 influences PILS5 and PILS6 abundance".

• Add scale bars to Figure 1B and 1D; Figure 2B, 2D, 2F, 2I, and 2K; Figure 3B and 3C; Figure 4A, 4C and 4G.

© 2022. Published by The Company of Biologists under the terms of the Creative Commons Attribution License (https://creativecommons.org/licenses/by/4.0/).

• Add target protein labels to the Western blots in Figure 3, Figure 4, Supplemental Figure 3, and Supplemental Figure 4.
• Add a "P274L" label to the gasp1-1 allele and SALK_091345 to the gasp1-2 allele on Supplemental Figure 2A.

Significance

• Nature and significance of the advance: this manuscript describes a novel genetic and biochemical regulatory module that may impact auxin transport and signaling in vivo to regulate primary root growth.

• Compare to existing published knowledge: this study is entirely novel and would deepen our understanding of PILS regulation and auxin regulatory loops.

• Audience: This manuscript will be of great interest to the auxin community and the Arabidopsis protein ubiquitination field. It is of general interest to those in the root biology field as well.

• Your expertise: I am an Assistant Professor who has been working on auxin pathway proteins for several years. I have expertise in plant ubiquitination analyses and hormone regulation. I have extensively used Arabidopsis as a model system and have expertise in root growth and development.

Reviewer 2

Evidence, reproducibility and clarity

Summary

The work by Feraru et al presents the results of a forward genetic screen aiming at uncovering modulators of the PILS6 overexpression phenotypes and PILS6 protein turnover in Arabidopsis thaliana. The authors identified GASP1, a RING/U box H2 protein as a protein involved in a post-transcriptional regulatory pathway leading to a reduced PIL6 abundance at the ER membrane. In total, 8 mutants with a weaker PILS6 signal and 13 mutants with a stronger PILS6 signal in roots of 35S:PILS6-GFP seedlings treated for 24h at 29oC were identified.

Major comments
The work seems preliminary and the authors have tendency to generalise or overstated the findings. The work is performed with two alleles and allelic test are convincing enough that the GASP1 locus is linked to the observed changes in the gasp1-1 background.
For example, the abstract should be more precise. The data concerns only PILS6 and PILS5. Reading the abstract lets the readers to think the identified regulation happens in all the PILS proteins. This has not been demonstrated. The same comment applies to the sentence line 92: "...suggesting that GAPS1 affects multiple PILS proteins in a variety of tissues". The data indicates that in gasp1 mutants, PILS5 and PILS6 abundance is reduced in roots. Phenotyping is performed only the roots and etiolated hypocotyls of young seedlings. Why only PILS5 was also tested? this is not well justified in the text.
My comments are suggestions how to improve the presented work.

All the experiments are performed using a constitutive promoter. Does GASP1 also affect endogenous PILS6 and PILS5? Is there any tissue or conditions when GASP1 and PILS6 are coexpressed in the same cell? Biological relevance is difficult to identify if only ectopic expression is analysed.

The authors tried to understand why GASP1 would regulate the abundance of PILS6. Being a RING2/U box protein suggests a function in proteasome-dependent protein degradation. The RING proteins are E3 ligase and may be involved in the transfer of ubiquitin to a protein targeted to degradation. But is GASP1 expected to bind its substrate directly or within a protein complex? Is there an additional domain, other than the RING H2 domain, that would suggest protein interaction with a substrate? Is it a functional E3 ligase? Is it binding to any E2? What is your hypothesis on this

© 2022. Published by The Company of Biologists under the terms of the Creative Commons Attribution License (https://creativecommons.org/licenses/by/4.0/).

complex? In the provided references, GASP1 is published to be an active E3 ligase, is PILS6 or PILS5 ubiquitinated? Did you identify any lysines that you would expect to be ubiquitinated?

The authors reached to the conclusion that GASP1 is indirectly affecting PILS6 abundance. Does GAPS1 mutation affect other ER proteins or only PILS? Using qPCR analysis and showing that some auxin-responsive genes are downregulated independently from PILS6, the authors concluded that GASP1 reduces PILS protein abundance (all PILS?) by repressing auxin signalling. Are there any other auxin responsive proteins or any other auxin transporters affected as well? For the MG132 and BTY treatment, was a control made with another ER-localized protein to ensure this is not a generic effect?

The regulation of the abundance of PILS is an argument in favor of the maintenance of auxin homeostasis in the nucleus. However, using the DR5 reporter only show the impact on the output of auxin signalling, not on active auxin levels and homeostasis. Please carefully revise the text in that sense. Degron-based reporters would provide you with the answer related to the homeostasis. Also, what is the DR5 pattern in the root tips (Figure 4)? The authors previously showed that DR5 fluorescence intensity is affected in PILS6oe. Is it also in gasp1 background?

Concerning GFP-GASP1, the authors stated that with the endogenous promoter no expression was observed without more details. Providing an expression profile would help to identify in which tissues and organs the GASP1 proteins are more abundant. Another hypothesis may be that the fusion is not functional. What is the rationale behind the N-terminal fusion? Is GFP-GASP1 functional? Also, what is the promoter used in figure 3B? If the GASP1 function is as E3 ligase, would it make sense to generate an inducible expression to avoid negative effects of an ectopic expression?

Minor comments
Please be more precise in the text, including the figure legends. The link between figure panels in main and supplemental figures should be more explicit when data are divided between the two (quantification and microscopic pictures) like between Figure 2 and S2.

Legend figure 1: what means "gloomy mutants were longer and shiny were shorter"? What is the size referring to?
Legend figure S2: "... positions of gasp1-1 and gasp1-2 mutants" should be "position of the SNP in gasp1-1 and T-DNA insertion in the gasp1-2 mutant."
Figure S2: any qPCR analysis in gasp1-1?
Legend Figure 2: The mutants are allelic, not alleles.
Legend Figure 3: GFP-GASP1: what is the promoter used? and n=? in D, any stats? any significance. If not significant, the legend should be amended.
Legend Figure S3 and S4: these are loading controls, not normalisation as no quantification was performed. Is the stability of PILS6 in WT and in gasp1 mutant significantly different?

Line 128: line 128: vascular tissue, not just vascular.
In lines 141 and 142: Figure S4A, not S1A.
In line 215 "if not differently stated" should be "unless stated differently"

All scale bars are missing!
And Fonseca de Lima, 2021 is missing from the references list.

There are no indications of the number of biological replicates. And when n is below 30, like in many root and hypocotyl length analysis, I find this is within the range of variability. Fig1C, n=15, 17, 1E, n=19, fig2C, n=15, fig S1, n=8 and so on.

Comments concerning the methods
Was gasp1-2 backcrossed? Any secondary insertions in the background?
Please do not refer to other papers in methods instead of describing the methods. Take the space to describe the method properly. No one wants to go through a chain of papers (especially if one does not have access to them), to realise the method is not well described. This concerns the temperature treatment, qPCR, protein extraction protocols.
Sequencing of the mutant population: How did you compare the two datasets? What software? How

© 2022. Published by The Company of Biologists under the terms of the Creative Commons Attribution License (https://creativecommons.org/licenses/by/4.0/).

many SNPs were identified? How did you validate besides second allele?
qPCR: What normalisation genes were used? Are their expression stable in the mutant backgrounds? What is the efficiency of the qPCR primers? What is the methods of analysis?
Generation of GFP-GASP1, what happens the lines are transformed? On which generation did you work and how did you select them?

Significance

This work advances the knowledge about the regulation of the PILS proteins. There is little knowledge available of these proteins beside being putative auxin transporters to the ER and their role in an auxin buffering mechanism to maintain homeostasis.
Performing a forward genetic screen was one of the approaches to obtain more information of the post translational regulation of PILS6. And GASP1 will certainly provide valuable information, so will the other identified mutants.

Reviewer 3

Evidence, reproducibility and clarity

Summary:
The authors identified GASP1 as a previously uncharacterized modulator of auxin signaling rates. GASP1 encodes an uncharacterized E3 ligase that belongs to the RING/U-box superfamily and supposedly mediates substrate-specific ubiquitination. The authors suggested that GASP1 does not directly interact with PILS proteins; rather, its impact on auxin signaling indirectly affects PILS turnover.

Major comments:
1. Line 28: "it (GASP1) indirectly lowers PILS protein abundance by repressing auxin signaling." However, the knocking out of GASP1 (gasp1-1 or gasp1-2) results in a decrease in auxin signaling and reduced PILS6-GFP level; therefore, the function of the protein is the opposite of what the authors propose: GASP1 enhances auxin signaling, which possibly enhances the PILS6-GFP level.
2. Line 28-29: "Our data suggests that low and high auxin conditions increase and reduce PILS protein, respectively." However, the application of the auxin biosynthesis inhibitor (KYN) decreased the PILS6-GFP signal, while the administration of external auxin (100 nM IAA) increased the PILS6-GFP signal. Thus, the effect of auxin level on PILS abundance is different from what is presented in the Abstract. In the Results and Discussion, the authors properly describe these events (see lines 143-144)
3. I feel that insufficient information is provided regarding how GASP1 affects the auxin response: does GASP1 regulate auxin synthesis, conjugation, or auxin signaling? I believe that the additional experiments in this direction (e.g., analysis of the expression of genes involved in auxin biosynthesis/catabolism) would further strengthen this paper.
4. There is no discussion about the effect of auxin on PILS. The data shows that auxin stabilizes PILS6-GFP. What is the possible mechanism? (This should be at least speculated in the discussion part.)
5. The motivation (methodology of mutant screening) for this work was supposedly the observation of thermo-sensitivity of PILS6 turnover; however, the authors did not come back to discuss this at the end of the story. It would be good to include it.

Significance

This paper is a continuation of the authors' previous work and includes further characterization of PILS proteins.

© 2022. Published by The Company of Biologists under the terms of the Creative Commons Attribution License (https://creativecommons.org/licenses/by/4.0/).

Author response to reviewers' comments

1. General Statements [optional]

This section is optional. Insert here any general statements you wish to make about the goal of the study or about the reviews.

We would like to thank the Editor and the three reviewers for their time dedicated to our MS. We highly appreciate the reviewers' constructive comments and recommendations that certainly improved our manuscript.

2. Point-by-point description of the revisions

This section is mandatory. Please insert a point-by-point reply describing the revisions that were already carried out and included in the transferred manuscript.

Reviewer #1 (Evidence, reproducibility and clarity (Required)):

Summary: This manuscript identifies a RING/U-box protein, named GASP1, as a putative novel regulator of PILS5 and PILS6 abundance and auxin response in Arabidopsis seedlings. The novel data is high quality and of great interest to the auxin and protein ubiquitination community.
We thank you for your encouraging words.

Major Comments:
• If GASP regulates (directly or indirectly) the degradation of PILS6 then one might expect increased levels of PILS6 in the absence of GASP. However, the opposite is observed (i.e. reduced levels of PILS6 in gasp1-1). In addition, the MG132 and BTZ treatments of PILS6OE lines indicate increased protein abundance. An alternative model might be that GASP-mediated ubiquitination of PILS6 is required for protein activation and/or subcellular localization, which has been reported for other eukaryotic ubiquitinated substrates. A working model for GASP and PILS would help synthesize the authors findings; perhaps this can be added as a final supplemental figure?
GASP1 is an uncharacterized gene with no function attributed thus far. Assuming that GASP1 is an H-type RING with E3 ubiquitin ligase activity (Stone et al. 2005 and Kraft et al., 2005), we agree with the reviewer that direct GASP1 targets are expected to be stabilized in the *gasp1* mutant. On the contrary, PILS6 and PILS5 show reduced abundance in *gasp1* mutants. Pharmacological inhibition of the proteasome reduced the turnover of PILS6, but this appeared largely independent of GASP1. Moreover, our data suggests that PILS proteins and GASP1 neither colocalize nor interact and, hence, we collectively propose that GASP1 indirectly controls the abundance of PILS proteins. Notably, we did not observe an apparent change in subcellular distribution of PILS5 or PILS6 in *gasp1* and hence we did not consider this further.
We improved the discussion on this matter and also provided a working model as suggested (Supplemental Figure 4F).

• The light regulated dynamics of GASP1 localization (Figure 3) is quite intriguing! In addition, the data shown in Figure 3B and 3C suggests that GASP and PILS6 could interact at the plasma membrane, perhaps via a complex.
We believe that the mentioned differences in the GASP1 localization in the light- and dark-grown seedlings rather relate to differences in cell morphology. Compared to light-grown hypocotyls, the dark-grown hypocotyls have very long and vacuolated cells, where the cytosol with the nucleus appear as a thin layer at the periphery of the cells. Hence, mentioned differences in GASP1 localization are likely due to these morphological aspects of the cell. To further consolidate that GASP1 is not ER-localized, we performed a colocalization with the ER-localized PILS3-RFP and analyzed the seedlings in F1, which is now presented in the Figure 3C of the revised manuscript.

• Can the authors speculate on the effects of the gasp1-1 allele and how the P274L mutation may impact GASP function? It is interesting that this allele and the null allele (gasp1-2) have the same genetic effect, but also a bit puzzling.
We performed a BLAST analysis and found that P274L mutation is not in the RING motif, but in a region that is not conserved among eukaryotic E3 ligases. Hence, we could not find any information about this amino acid. However, it's known from the literature that Proline to Leucine substitutions cause abnormal conformational and structural changes that affect dramatically the protein function (Vilson et al., 1989; Molnar et al., 2016). We improved the depiction of this mutation in the revised version.

© 2022. Published by The Company of Biologists under the terms of the Creative Commons Attribution License (https://creativecommons.org/licenses/by/4.0/).

Minor Comments:

• Previous work has described and demonstrated that PILS proteins are auxin "transporters", which is a bona fide protein functional category. Use of the term auxin "facilitator" (lines 22, 39 and 145) may be confusing for readers as it is not technically a protein functional category. The PILS proteins may be described as facilitating transport, but I would caution against using this term as a noun.
Auxin transport assays at the plasma membrane is relatively straight forward. It is however challenging to perform auxin transport assays for ER retained transporters. Here, auxin transport can only indirectly be assessed, because labelled auxin first needs to enter the cell and may subsequently be modified before reaching the ER membrane. Accordingly, previous data showed that PILS proteins define the intracellular accumulation of auxin (Barbez et al., 2012) and reduce the nuclear abundance of auxin (Feraru et al., 2019), but transporter activity (in a stricter sense) remains to be addressed. Accordingly, the term “putative auxin carrier” or “intracellular auxin transport facilitator” is currently more appropriate.

• In the Cloning section of the methods, line 226, please add the concentration of kanamycin used for transgenic selection.
We added it.

• Figure 1A legend: suggest modifying the sentence "Overall, the gloomy mutants were longer and the shiny mutants were shorter" (lines 339-340) to "Overall, the gloomy mutant roots were longer and the shiny mutant roots were shorter".
We replaced by “Overall, the *gloomy* and *shiny* mutants displayed enhanced and reduced growth when compared to PILS6ox, respectively.” because it was not only the root, but also the cotyledons were different.

• Figure 2I-L legend: suggest modifying the title "GASP1 is not specific to PILS6" (line 363) to "GASP1 influences PILS5 and PILS6 abundance".
Because the data relates only to PILS5, we changed by “*gasp1-2* affects *PILS5*^{OE} hypocotyl phenotype and PILS5-GFP fluorescence.”

• Add scale bars to Figure 1B and 1D; Figure 2B, 2D, 2F, 2I, and 2K; Figure 3B and 3C; Figure 4A, 4C and 4G.
Scale bars were added to all images.

• Add target protein labels to the Western blots in Figure 3, Figure 4, Supplemental Figure 3, and Supplemental Figure 4.
The bands were marked with asterisk and mentioned in Figure legends.

• Add a "P274L" label to the gasp1-1 allele and SALK_091345 to the gasp1-2 allele on Supplemental Figure 2A.
Information was added.

Reviewer #1 (Significance (Required)):

• Nature and significance of the advance: this manuscript describes a novel genetic and biochemical regulatory module that may impact auxin transport and signaling in vivo to regulate primary root growth.

• Compare to existing published knowledge: this study is entirely novel and would deepen our understanding of PILS regulation and auxin regulatory loops.

• Audience: This manuscript will be of great interest to the auxin community and the Arabidopsis protein ubiquitination field. It is of general interest to those in the root biology field as well.

• Your expertise: I am an Assistant Professor who has been working on auxin pathway proteins for several years. I have expertise in plant ubiquitination analyses and hormone regulation. I have extensively used Arabidopsis as a model system and have expertise in root growth and development.
We thank you for your comments/suggestions and encouraging words on our work.

© 2022. Published by The Company of Biologists under the terms of the Creative Commons Attribution License (https://creativecommons.org/licenses/by/4.0/).

Reviewer #2 (Evidence, reproducibility and clarity (Required)):

Summary
The work by Feraru et al presents the results of a forward genetic screen aiming at uncovering modulators of the PILS6 overexpression phenotypes and PILS6 protein turnover in Arabidopsis thaliana. The authors identified GASP1, a RING/U box H2 protein as a protein involved in a post-transcriptional regulatory pathway leading to a reduced PIL6 abundance at the ER membrane. In total, 8 mutants with a weaker PILS6 signal and 13 mutants with a stronger PILS6 signal in roots of 35S:PILS6-GFP seedlings treated for 24h at 29oC were identified.

Major comments
The work seems preliminary and the authors have tendency to generalise or overstated the findings. The work is performed with two alleles and allelic test are convincing enough that the GASP1 locus is linked to the observed changes in the gasp1-1 background.
For example, the abstract should be more precise. The data concerns only PILS6 and PILS5. Reading the abstract lets the readers to think the identified regulation happens in all the PILS proteins. This has not been demonstrated.
We indeed only show that PILS5 and PILS6 levels are affected by the identified *gasp1* and it remains to be seen if other PILS proteins behave the same or deviate. We followed your suggestion and removed throughout the manuscript the general statements on PILS protein regulations.

The same comment applies to the sentence line 92: "...suggesting that GAPS1 affects multiple PILS proteins in a variety of tissues".
We specified our conclusions.

The data indicates that in gasp1 mutants, PILS5 and PILS6 abundance is reduced in roots. Phenotyping is performed only the roots and etiolated hypocotyls of young seedlings. Why only PILS5 was also tested? this is not well justified in the text.
My comments are suggestions how to improve the presented work.
Compared to PILS6, PILS5 overexpression causes a stronger phenotype in dark grown hypocotyls. We intended to include data from both dark and light-grown seedlings and therefore focused on both PILS5 and PILS6. For clarity, we modified the text accordingly.

All the experiments are performed using a constitutive promoter. Does GASP1 also affect endogenous PILS6 and PILS5?
Various stimuli, including auxin, brassinosteroid, light, and temperature, impact on the transcription and turnover of PILS proteins (Barbez et al., 2012; Beziat et al., 2017; Feraru et al., 2019; Sun et al., 2019). Here we aimed to address specifically the turnover of PILS proteins and therefore focused on the constitutive promoter. In this manuscript, we uncovered the PILS-dependent auxin feedback mechanism on its own signaling output. Upcoming work will further deepen our understanding of the developmental importance of this mechanism.

Is there any tissue or conditions when GASP1 and PILS6 are coexpressed in the same cell? Biological relevance is difficult to identify if only ectopic expression is analysed.
In this MS we do not focus on the biological role of *GASP1*, but rather focused on the unexpected finding that *gasp1* mutants show severely defective auxin signaling output without a major impact on auxin related phenotypes. Accordingly, we assume that the PILS-dependent compensation mechanism disguises the developmental role of GASP1.
GASP1 seems however to be ubiquitously expressed (Klepikova et al., 2016) and it was identified as a mobile transcript (root-to-shoot) (Thieme et al., 2015), so GASP1 could function also as a messenger RNA in distant tissues. However, the in-depth elucidation of GASP1 function in plant development is beyond the scope of this manuscript.

The authors tried to understand why GASP1 would regulate the abundance of PILS6. Being a RING2/U box protein suggests a function in proteasome-dependent protein degradation. The RING proteins are E3 ligase and may be involved in the transfer of ubiquitin to a protein targeted to degradation. But is GASP1 expected to bind its substrate directly or within a protein complex? Is there an additional domain, other than the RING H2 domain, that would suggest protein interaction with a substrate? Is it a functional E3 ligase? Is it binding to any E2? What is your hypothesis on this

© 2022. Published by The Company of Biologists under the terms of the Creative Commons Attribution License (https://creativecommons.org/licenses/by/4.0/).

complex? In the provided references, GASP1 is published to be an active E3 ligase, is PILS6 or PILS5 ubiquitinated? Did you identify any lysines that you would expect to be ubiquitinated?
These are all very good questions to understand the biological function of GASP, which however goes beyond the scope of this manuscript. Previous research (Stone et al. 2005 and Kraft et al., 2005) indeed proposed that GASP1 has E3 ligase activity, which seems related to the E2 ligases UBC8 and UBC11 (Kraft et al., 2005). Besides the RING H2 domain, our alignments of GASP1 did not show any other conserved domains. We have identified lysine sites in the central cytosolic loop region of all *PILS* genes, the highest number being identified in PILS5 (10 K). We envision that the PILS proteins are ubiquitinated at some of these sites, but not directly by GASP1.

The authors reached to the conclusion that GASP1 is indirectly affecting PILS6 abundance. Does GAPS1 mutation affect other ER proteins or only PILS? Using qPCR analysis and showing that some auxin-responsive genes are downregulated independently from PILS6, the authors concluded that GASP1 reduces PILS protein abundance (all PILS?) by repressing auxin signalling. Are there any other auxin responsive proteins or any other auxin transporters affected as well? For the MG132 and BTY treatment, was a control made with another ER-localized protein to ensure this is not a generic effect?
We assume that GASP1 modulates auxin signaling output, which in turn impacts on PILS proteins turnover. To address this question, we included the ER maker DERLIN1 fused with mScarlet. The in- and decrease of auxin levels did not affect the DER1-mScarlet abundance. We accordingly assume that the auxin effect on PILS proteins shows certain specificity and does not impact all ER resident proteins. Similarly, the DER1-mScarlet marker was also not affected by the pharmacological inhibition of the proteasome. Again, assuming that the effect on PILS reflects certain specificity. We included this set of data (Figures 3F, 3G and Supplementary Figures 4D, 4E) in the revised version of this manuscript.

The regulation of the abundance of PILS is an argument in favor of the maintenance of auxin homeostasis in the nucleus. However, using the DR5 reporter only show the impact on the output of auxin signalling, not on active auxin levels and homeostasis. Please carefully revise the text in that sense. Degron-based reporters would provide you with the answer related to the homeostasis.
We revised the text as suggested, focusing on auxin signaling output.

Also, what is the DR5 pattern in the root tips (Figure 4)? The authors previously showed that DR5 fluorescence intensity is affected in PILS6oe. Is it also in gasp1 background?
In contrast to the dramatic reduction of DR5::GFP signal in the dark-grown hypocotyls and in the upper region of the root, DR5::GFP signal intensity in the very tip of the root is similar in WT and *gasp1-2* mutant. We added this information in the text and Supplementary Figures 4A, 4B.

Concerning GFP-GASP1, the authors stated that with the endogenous promoter no expression was observed without more details. Providing an expression profile would help to identify in which tissues and organs the GASP1 proteins are more abundant. Another hypothesis may be that the fusion is not functional. What is the rationale behind the N-terminal fusion? Is GFP-GASP1 functional?
We used C- and N- terminal fusion in the transgenic GASP1::GASP1-GFP and 35S::GFP-GASP1, respectively. We hence cannot conclude whether the promoter or fusion protein limits its detection. We hence removed the statement from the revised manuscript. While detectable, 35S::GFP-GASP1 does not induce an obvious phenotype and we currently do not know if the fusion is fully functional.

Also, what is the promoter used in figure 3B?
We used a *35S* promoter.

If the GASP1 function is as E3 ligase, would it make sense to generate an inducible expression to avoid negative effects of an ectopic expression?
We did not notice any obvious morphological phenotypes in our lines overexpressing *GASP1*.

Minor comments
Please be more precise in the text, including the figure legends. The link between figure panels in main and supplemental figures should be more explicit when data are divided between the two

© 2022. Published by The Company of Biologists under the terms of the Creative Commons Attribution License (https://creativecommons.org/licenses/by/4.0/).

(quantification and microscopic pictures) like between Figure 2 and S2.
We improved the figure depiction for better readability.

Legend figure 1: what means "gloomy mutants were longer and shiny were shorter"? What is the size referring to?
These mutants are in the PILS6 overexpression background and hence the statements refer to this background. We replaced by “Overall, the *gloomy* and *shiny* mutants displayed enhanced and reduced growth when compared to PILS6ox, respectively.”

Legend figure S2: "... positions of gasp1-1 and gasp1-2 mutants" should be "position of the SNP in gasp1-1 and T-DNA insertion in the gasp1-2 mutant."
We corrected it.

Figure S2: any qPCR analysis in gasp1-1?
The transcription of *GASP1* was not altered in this allele. We included a new qPCR with the both alleles in the Supplementary Figure 2B.

Legend Figure 2: The mutants are allelic, not alleles.
We corrected it.

Legend Figure 3: GFP-GASP1: what is the promoter used? and n=? in D, any stats? any significance. If not significant, the legend should be amended.
We used a 35S promoter. This information has been added. The ratios presented in D (now E) were calculated with the values from the graph shown in Supplementary Figure 3G, which also depicts the statistical evaluation and sample size information. For clarity, we also moved the detailed graph from the Supplementary Figure 3 (G) to the Figure 3 (E).

Legend Figure S3 and S4: these are loading controls, not normalisation as no quantification was performed. Is the stability of PILS6 in WT and in gasp1 mutant significantly different?
We performed normalizations and quantified band intensities. We added the values on the images from Figures 3A and 4I.

Line 128: line 128: vascular tissue, not just vascular.
In lines 141 and 142: Figure S4A, not S1A.
In line 215 "if not differently stated" should be "unless stated differently"
We corrected all.

All scale bars are missing!
We added all missing scale bars.

And Fonseca de Lima, 2021 is missing from the references list.
It was inserted at position 6, because of incorrect abbreviation. We corrected it.

There are no indications of the number of biological replicates. And when n is below 30, like in many root and hypocotyl length analysis, I find this is within the range of variability. Fig1C, n=15, 17, 1E, n=19, fig2C, n=15, fig S1, n=8 and so on.
We did more than three replicates and they are all reproducible. We included a general statement in the revised version of the manuscript. We grow all seedlings used for one particular experiment (confocal imaging, phenotyping, etc.) just on one single line on the plate (same height). Thereby, they are all exposed to the same light intensity and humidity in the plate. When the seedlings are exposed to the same microenvironment in the plate (including control and mutants), the variability among the seedlings is particularly low. While it is beneficial to balance “the range of variability”, it obviously limits the number of seedlings that can be grown alongside in the same plate.

Comments concerning the methods
Was gasp1-2 backcrossed? Any secondary insertions in the background?
We backcrossed the EMS mutant once, but not the salk t-DNA insertion.

Please do not refer to other papers in methods instead of describing the methods. Take the space to describe the method properly. No one wants to go through a chain of papers (especially if one

© 2022. Published by The Company of Biologists under the terms of the Creative Commons Attribution License (https://creativecommons.org/licenses/by/4.0/).

does not have access to them), to realise the method is not well described. This concerns the temperature treatment, qPCR, protein extraction protocols.
We described the methods.

Sequencing of the mutant population: How did you compare the two datasets? What software? How many SNPs were identified? How did you validate besides second allele?
We improved the used methodology. Besides crossing PILS6-GFP to a second allele, we used allelic test in F1 to validate the causal gene.

qPCR: What normalisation genes were used? Are their expression stable in the mutant backgrounds? What is the efficiency of the qPCR primers? What is the methods of analysis?
We used Actin2 gene to normalize the qPCR data. This information is now provided in the material and method section.

Generation of GFP-GASP1, what happens the lines are transformed? On which generation did you work and how did you select them?
Segregating F2 were selected for Kanamycin resistant and presence of fluorescence. We worked with non-segregating, F3 homozygous lines. We provided this information in the revised manuscript.

Reviewer #2 (Significance (Required)):

This work advances the knowledge about the regulation of the PILS proteins. There is little knowledge available of these proteins beside being putative auxin transporters to the ER and their role in an auxin buffering mechanism to maintain homeostasis.
Performing a forward genetic screen was one of the approaches to obtain more information of the post translational regulation of PILS6. And GASP1 will certainly provide valuable information, so will the other identified mutants.
We thank you for your helpful comments and suggestions.

Reviewer #3 (Evidence, reproducibility and clarity (Required)):

Summary:
The authors identified GASP1 as a previously uncharacterized modulator of auxin signaling rates. GASP1 encodes an uncharacterized E3 ligase that belongs to the RING/U-box superfamily and supposedly mediates substrate-specific ubiquitination. The authors suggested that GASP1 does not directly interact with PILS proteins; rather, its impact on auxin signaling indirectly affects PILS turnover.

Major comments:
1. Line 28: "it (GASP1) indirectly lowers PILS protein abundance by repressing auxin signaling." However, the knocking out of GASP1 (gasp1-1 or gasp1-2) results in a decrease in auxin signaling and reduced PILS6-GFP level; therefore, the function of the protein is the opposite of what the authors propose: GASP1 enhances auxin signaling, which possibly enhances the PILS6-GFP level.
2. Line 28-29: "Our data suggests that low and high auxin conditions increase and reduce PILS protein, respectively." However, the application of the auxin biosynthesis inhibitor (KYN) decreased the PILS6-GFP signal, while the administration of external auxin (100 nM IAA) increased the PILS6-GFP signal. Thus, the effect of auxin level on PILS abundance is different from what is presented in the Abstract. In the Results and Discussion, the authors properly describe these events (see lines 143-144)
We thank you for pinpointing these mistakes. The misleading information resulted from erroneous editing of the abstract. We corrected it.

3. I feel that insufficient information is provided regarding how GASP1 affects the auxin response: does GASP1 regulate auxin synthesis, conjugation, or auxin signaling? I believe that the additional experiments in this direction (e.g., analysis of the expression of genes involved in auxin biosynthesis/catabolism) would further strengthen this paper.
We observed that loss of *GASP1* function had a dramatic effect on auxin signaling output (DR5::GFP and auxin responsive genes), but surprisingly this did not relate to strong phenotypic deviations.

© 2022. Published by The Company of Biologists under the terms of the Creative Commons Attribution License (https://creativecommons.org/licenses/by/4.0/).

This prompted us to focus here on the underlying compensatory mechanism. Upcoming work should indeed address the biological role of GASP1, which however exceeds the scope of this manuscript.

4. There is no discussion about the effect of auxin on PILS. The data shows that auxin stabilizes PILS6-GFP. What is the possible mechanism? (This should be at least speculated in the discussion part.)
We thank you for this suggestion and included now some discussion on the possible mechanism.

5. The motivation (methodology of mutant screening) for this work was supposedly the observation of thermo-sensitivity of PILS6 turnover; however, the authors did not come back to discuss this at the end of the story. It would be good to include it.
We followed your suggestions and discussed it in the revised manuscript.

Reviewer #3 (Significance (Required)):
This paper is a continuation of the authors' previous work and includes further characterization of PILS proteins.
We thank you for your helpful comments and suggestions.

Original submission

First decision letter

MS ID#: DEVELOP/2022/200929

MS TITLE: PILS proteins provide a homeostatic feedback on auxin signaling output

AUTHORS: Elena Feraru, Mugurel I Feraru, Jeanette Moulinier-Anzola, Maximilian Schwihla, Jonathan Ferreira Da Silva Santos, Lin Sun, Sascha Waidmann, Barbara Korbei, and Jurgen Kleine-Vehn
ARTICLE TYPE: Research Article

Thank you for sending your manuscript to Development through Review Commons.

I am happy to tell you that your manuscript has been accepted for publication in Development, pending our standard ethics checks.

Reviewer 1

Advance summary and potential significance to field

This manuscript identified the RING protein GASP1 as an indirect regulator of PILS5 and PILS6 proteins stability. GASP1 was identified in a forward genetic screen for changes in GFP intensity in the 35S:PILS6-GFP thus focusing on the translational regulation of PILS6. In that respect, GASP1 is a good candidate. Even so the function of GASP1 is largely unknown, and its study beyond the scoop of this study, this work brings some information of the links between nuclear auxin signaling, auxin homeostasis, auxin transport to the ER (and buffering from the nuclear pool), and the PILS protein.

Comments for the author

This revised manuscript is less speculative than the previous version. All the text is now supported by the data. And the authors responded to the comments made with the initial version in Review Commons.
Therefore, I would only minor comments
1> In line 57, you mentioned that "the compartmentalization of auxin into ER lumen correlates with higher auxin conjugation rates". A reference to the work showing this is missing. Indeed, in Včelařová, L. et al. Auxin Metabolome Profiling in the Arabidopsis Endoplasmic Reticulum Using an

© 2022. Published by The Company of Biologists under the terms of the Creative Commons Attribution License (https://creativecommons.org/licenses/by/4.0/).

Optimised Organelle Isolation Protocol. Int J Mol Sci 22, 9370 (2021), the authors isolated ER and showed that ER contains more free IAA than cellular extracts. This is not contradictory with your statement. But was it shown?
2> Line 200: remove "for". It is "encodes an uncharacterised E3 ubiquitin ligase" or "codes for an..."
3> Line 270, please indicate the name of the company used for sequencing.
4> the qPCR analysis requires more description. Was the ACTIN2 expression identical in the genotypes? Were the primers tested prior the reaction (efficiency, dimers etc)? Was is the method used for the comparison?
The authors need to follow the MIQE guidelines, and it is now not reflected in the method description.

Reviewer 2

Advance summary and potential significance to field

The authors identified GASP1 as a previously uncharacterized modulator of auxin signaling rates. GASP1 encodes an uncharacterized E3 ligase that belongs to the RING/U-box superfamily and supposedly mediates substrate-specific ubiquitination.
The authors suggested that GASP1 does not directly interact with PILS proteins; rather, its impact on auxin signaling indirectly affects PILS turnover.
The work is of great interest to auxin community

Comments for the author

The work is of a significant contribution to auxin community

Reviewer 3

Advance summary and potential significance to field

In this manuscript, the identification of a novel gasp mutant that can diminish expression of a fluorescently tagged PILS6 protein has uncovered a new mode of posttranslational gene regulation for a family of auxin efflux carriers. In addition, microscopic and pharmacological studies on the feedback between auxin biosynthesis, auxin signaling, and auxin transport provide new knowledge into the potential links between these molecular pathways.

The mechanistic insight into how GASP influences PILS5 and PILS6 abundance in vivo still remains, as well as how the P>L mutation in gasp1 affects function of this putative E3 ligase. These studies are worthy of future studies (but beyond the scope of this manuscript).

Comments for the author

1. Numerous E3 ubiquitin ligases do not direct ubiquitin-mediated degradation of substrate proteins. The data presented in this work suggest that GASP1 is not required for the degradation, but rather the stability of PILS6 and PILS5. The authors may want to consider this alternative hypothesis which is more consistent with their data and re-write lines 150-152.
2. Line 185: suggest removing “and hence signaling”. This experiment is focused on altering biosynthesis, not signaling per se.
3. Figure 4E & F – denote statistical significance on measured gene expression values as warranted.
4. Please add a section listing the accession numbers for the genes in the study (GASP, PILS6, PILS5 and PILS3) to the Materials and Methods section.
5. Change qRT-PCR (line 327) to RT-qPCR (the PCR assay is quantitative, not the reverse transcription reaction).

© 2022. Published by The Company of Biologists under the terms of the Creative Commons Attribution License (https://creativecommons.org/licenses/by/4.0/).
